# Does intracostal suture placement during closure reduce pain post-thoracotomy compared to routine pericostal closure?

**DOI:** 10.1186/1749-8090-10-S1-A192

**Published:** 2015-12-16

**Authors:** Sanjeet SA Singh, Helen Monaghan, Alan JB Kirk

**Affiliations:** 1 Golden Jubilee National Hospital, Clydebank, Dumbartonshire, G81 4DY, UK

## Background/Introduction

Postero-lateral thoracotomies are routinely performed in thoracic surgery. Post-operatively, the pain can be incapacitating due to many factors including intercostal nerve injury. Therefore neurovascular bundle (NVB) protection may be a key element in improving pain control. Two methods of chest closure are used in our unit; traditional pericostal closure and intracostal closure.

## Aims/Objectives

To investigate whether intracostal suture placement reduces acute postoperative thoracotomy pain and length of stay.

## Method

A retrospective cohort study involving 1031 patients who underwent primary postero-lateral thoracotomies in the past 3 years was conducted at our regional cardiothoracic centre.

5 different surgeons, 4 using pericostal sutures and 1 intracostal sutures placement performed the surgeries. Patient's primary method of analgesia was per anaesthetists' preference.

After completion of the appropriate procedure, patients had one or two soft chest tubes placed as per surgeon's preference.

In the pericostal group, Vicryl 2.0 sutures were placed around their ribs in the standard pericostal fashion.

The intracostal group had their thoracotomies closed by drilling approximately four evenly spaced holes using a 5-mm attachment to a pneumatic drill. Vicryl 2.0 sutures were placed through the hole and just over the top of the rib above

Chest tubes were removed as per surgeons' preference. A visual analogue scale objectified pain at post op day 0, 1, 2 and 3. Pain scores and length of admission were outcomes measured.

## Results

There were 744 patients in the pericostal group and 287 patients in the intracostal group. Patients in the intracostal group were younger (63 ± 12.3 years vs 66 ± 10.5, p = 0.01), but all other parameters were similar. The mean pain score for the pericostal group on post op day 3 and length of stay was significantly higher.

## Discussion/Conclusion

Intracostal suture placement reduces pain on post op day 3 and length of stay.

